# Antimicrobial Consumption and Susceptibility of *Neisseria gonorrhoeae*: A Global Ecological Analysis

**DOI:** 10.3389/fmed.2018.00329

**Published:** 2018-11-27

**Authors:** Chris Kenyon, Jozefien Buyze, Teodora Wi

**Affiliations:** ^1^Sexually Transmitted Infections HIV/STI Unit, Institute of Tropical Medicine, Antwerp, Belgium; ^2^Division of Infectious Diseases and HIV Medicine, University of Cape Town, Cape Town, South Africa; ^3^Clinical Trials Unit, Institute of Tropical Medicine, Antwerp, Belgium; ^4^Department of Reproductive Health and Research, World Health Organization, Geneva, Switzerland

**Keywords:** *N. gonorrhoeae*, antimicrobial resistance, antibiotic consumption, susceptibility, Global Warming

## Abstract

**Aims:** The reasons why antimicrobial resistance in *Neisseria gonorrhoeae* has emerged explosively in certain populations but not others are poorly understood. We hypothesized that population level consumption of antimicrobials plays a role.

**Methods:** Using susceptibility data from the World Health Organizations Global Gonococcal Antimicrobial Surveillance Programme and antimicrobial consumption data from the IMS Health MIDAS database we built linear regression models with country-level cephalosporin, macrolide, and fluoroquinolone consumption (standard doses/1,000 population/year) as the explanatory variable and 1-year lagged ceftriaxone, azithromycin, and ciprofloxacin resistance as the outcome variables. These were performed at two time points 2008/2009 and 2013/2014.

**Results:** The association between antimicrobial resistance and consumption at the level of individual countries was positive in all six assessments. In four instances the positive associations were statistically significant (cephalosporins 2008: coefficient 0.0005 [95% confidence interval (CI) 0.0002–0.0007] and 2013: coefficient 0.0003 [95% CI 0.0002–0.0004]; macrolides 2013: coefficient 0.0005 [95% CI 0.00002–0.001]; fluoroquinolones 2013: coefficient 0.02 [95% CI 0.006–0.031]).

**Conclusions:** Differences in population level consumption of particular antimicrobials may play a role in explaining the variations in the emergence of antimicrobial resistance in *N. gonorrhoeae*.

## Background

Recent reports of combined high level resistance to ceftriaxone and azithromycin in *N. gonorrhoeae* (Ng) suggest that untreatable gonorrhea may not be far off (1). To prevent this we need to optimally understand the underlying determinants of antimicrobial resistance (AMR) and why AMR has emerged in certain populations but not others. An array of *in vitro* and individual-level studies have established a link between antimicrobial exposure and the development of AMR (2–8). These individual level studies have not however explained the striking geographic and social clustering in the emergence of AMR (5, 9–11). AMR has been noted to frequently first emerge in core-groups (sex workers or men who have sex with men [MSM]) but more often in core-groups in particular locales (5, 10). In the USA for example AMR has typically first emerged in MSM in the western states and then spread to the rest of the country (5, 12). The Western Pacific has also been disproportionately affected by AMR. Resistance to extended-spectrum cephalosporins (hence termed cephalosporins) was first documented in Japan in 2000 (7). By 2001 a third of Ng isolates exhibited decreased sensitivity to cefixime (13). The spread of these resistant Ng strains to other countries has been extensively documented and is one explanation for the frequent emergence of AMR on the West coast of the United (5, 12). What remains incompletely explained is why AMR to cephalosporins appeared first in Japan when there was little difference in the Ng treatment algorithm in Japan compared to elsewhere (13).

For a number of bacteria, striking ecological correlations have been found between the prevalence of antimicrobial consumption and resistance to that antimicrobial (14–17). In this study we assess for the first time the association between antimicrobial consumption and AMR in Ng at the level of world regions and countries.

## Methods

Our data came from three sources.

### Antimicrobial resistance

The WHO Global Gonococcal Antimicrobial Surveillance Programme (GASP) is a collaborative global network of regional and subregional reference laboratories that monitors AMR in Ng around the world. WHO GASP data have informed revisions of global, regional and national gonorrhea treatment guidelines, as well as public health strategies and policies developed by WHO and other organizations. The full GASP methodology, including suggested sampling strategy, laboratory techniques, external quality assurance, and internal quality control mechanisms has been published elsewhere (11). The following minimum inhibitory concentration (MIC) breakpoints are used to define antimicrobial resistance in GASP: Azithromycin (a macrolide): ≥1 μg/ml, Cefixime (a cephalosporin): >0.25 μg/ml, Ceftriaxone (a cephalosporin): >0.125 μg/ml, Ciprofloxacin (a fluoroquinolone): ≥1 μg/ml (11).

The total numbers of countries contributing AMR data to GASP increased from 56 in 2009 to 77 in 2014. These are divided into 6 WHO regions (Supplementary Table 1). We excluded the three countries from the Eastern Mediterranean Region as the number of countries from this region was small, they reported a low number of AMR prevalence estimates and there was only one country from this region with antimicrobial consumption data available. The list of these countries by WHO region and year of reporting is available in Supplementary Table 1.

### Antimicrobial consumption

Data from Intercontinental Marketing Statistics Health MIDAS (IMS Health, Danbury, CT, USA) were used as a measure of national antimicrobial drug consumption. IMS uses national sample surveys that are performed by pharmaceutical sales distribution channels to estimate antimicrobial consumption from the volume of antibiotics sold in retail and hospital pharmacies. The sales estimates from this sample are projected with use of an algorithm developed by IMS Health to approximate total volumes for sales and consumption. Antimicrobial consumption estimates are reported as the number of standard doses (a dose is classified as a pill, capsule, or ampoule) per 1,000 population per year. Data is available for 63 countries. We used data for the 47 countries that were also represented in the GASP dataset. Data was available from 45 countries in 2000 and 47 in 2008 and 2013.

### Incidence of Ng in 2012

The Ng incidence by WHO world region were taken from a publication that reported the WHOs estimated Ng incidence and prevalence (18). The Ng incidence rates were reported as rates per 1,000 population for 2012. The incidence rates were only reported by each gender separately.

### Data analysis

#### Regional level

Pearson's correlation was used to assess the association between median antimicrobial consumption (within each WHO Region) in 2008 and the median prevalence of AMR in the year 2009. This was then repeated for antimicrobial consumption in 2013 and AMR in 2014. In the case of ceftriaxone, the AMR data for 2013 was used instead of that of 2014 due to the higher number of data points in 2013 (*n* = 44) than 2014 (*n* = 22). Pearson's correlation was also used to assess the association between the estimated incidence of Ng in 2012 and (1) AMR in 2012 and (2) antimicrobial consumption in 2013. In both cases these analyses were done stratified by antimicrobial class and gender as the combined male/female incidence rates were not reported in the source document. Due to the small sample size (*n* = 5 regions), *p*-values are not reported. A Pearson's correlation coefficient above 0.3 or below −0.3 is defined as a positive or negative correlation. Differences in the prevalence of AMR between regions were assessed using the Kruskal Wallis test. If statistically significant differences were found, the Wilcoxon rank-sum test was used to test if there was a significant difference in prevalence between each region and the region of Europe.

#### Country level

We performed linear regression analyses in which the dependent variable was antimicrobial susceptibility (percent resistant) in each country and the independent variable of interest was the prior year antimicrobial consumption rate. The AMR variables used in these regressions were square root transformed to more closely approximate normal distributions.

Stata 13.0 was used for all analyses. A *p*-value of < 0.05 was considered statistically significant.

## Results

### Antimicrobial resistance

#### Ceftriaxone

**Figure 1:**
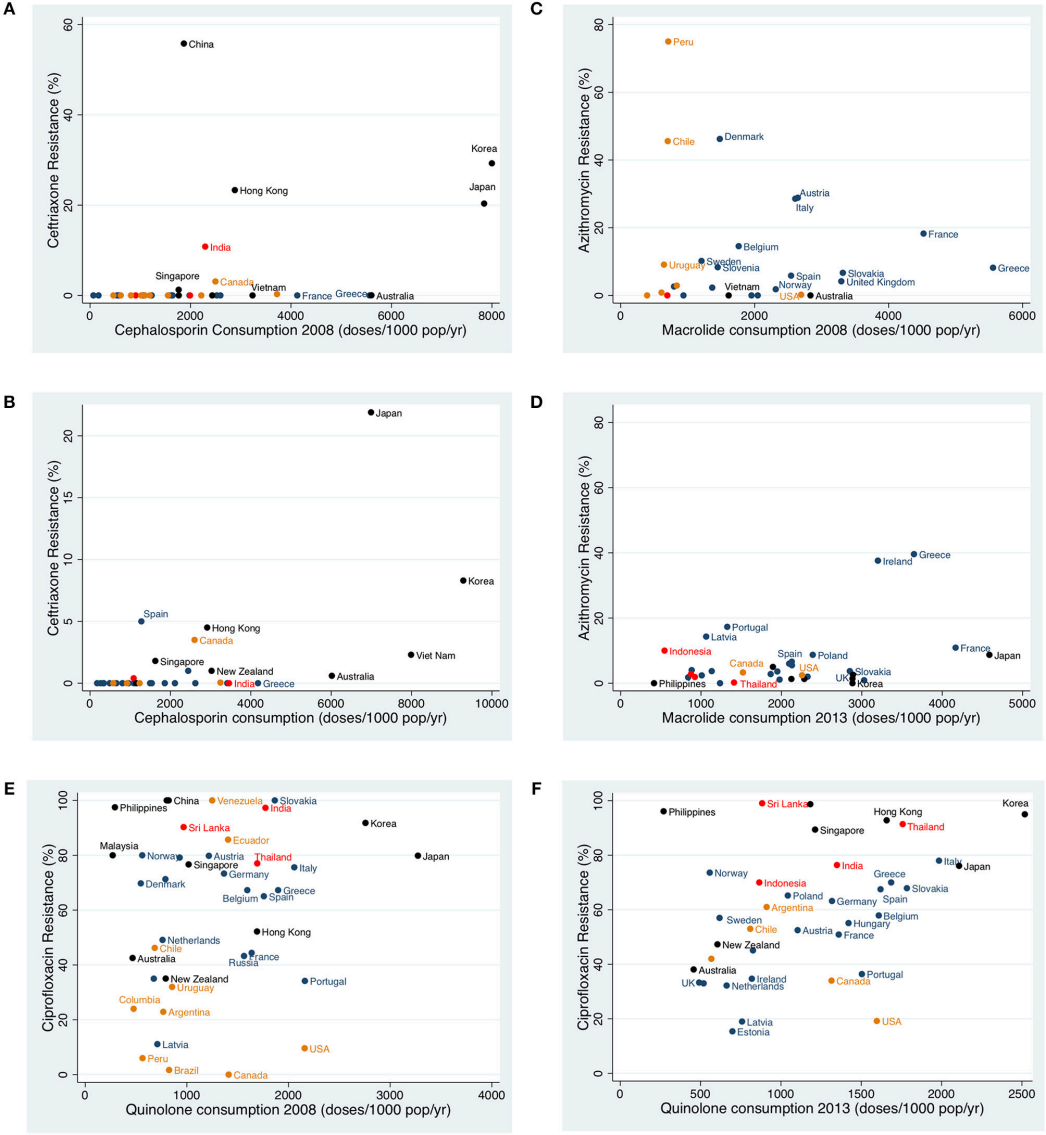
Scatter plots of antimicrobial consumption vs. antimicrobial resistance in the subsequent year for **(A)** ceftriaxone in 2008 **(B)** ceftriaxone in 2013 **(C)** azithromycin in 2008 **(D)** azithromycin in 2013 **(E)** ciprofloxacin in 2008 **(F)** ciprofloxacin in 2013 (Black, Western Pacific; Red, South East Asia; Yellow, Americas; Blue, Europe).

In both 2009 and 2013, the prevalence of ceftriaxone resistance was highest in the Western Pacific (median percent AMR: 1.3%, interquartile range [IQR] 0.0–20.3). These prevalence estimates, as well as that from South East Asia in 2009 (0.0, IQR 0.0–5.4) were higher than those in Europe at the same times (0.0, IQR 0.0–0.0, *p* < 0.05, Table 1, Figure 1).

**Table 1:** Antimicrobial resistance prevalence by country reported in world regions [median (interquartile range)] Data from GASP.

		**2009**	***N***	**2014**	***N***
Ceftriaxone	Europe	0.0 (0.0–0.0)	19	0.0 (0.0–0.0)	22
	SE Asia	0.0 (0.0–5.4)^*^	4	0.0 (0.0–0.2)	2
	W Pacific	1.3 (0.0–20.3)^***^	13	1.4 (0.2–4.5)^***^	10
	Americas	0.0 (0.0–1.3)	13	0.0 (0.0–0.3)	8
	Africa	0.0 (0.0–0.7)^*^	3	0.0 (0.0–0.0)	1
	Total	0.0 (0–0.016)^*^	52	0.0 (0.0–0.3)^*^	43
Azithromycin	Europe	6.7 (2.3–17.3)	19	3.7 (1.8–8.7)	23
	SE Asia	0.0 (0.0–0.0)	1	2.4 (0.2–4.8)	6
	W Pacific	0.0 (0.0–0.0)^*^	3	1.4 (1.3–5.0)	9
	Americas	2.9 (0.2–45.6)	7	2.9 (2.5–3.3)	2
	Africa	1.4 (0.0–2.7)	2	11 (10–83.1)	3
	Total	3.5 (0.0–12.4)^*^	32	3.45 (1.3–8.7)	43
Ciprofloxacin	Europe	67.3 (43.3–79.2)	19	57 (34.7–67.5)	23
	SE Asia	93.2 (83.7–96.7)^*^	4	91.1 (76.4–99.1)^**^	6
	W Pacific	75.7 (38.8–92.4)	16	82.8 (38.1–95.0)	10
	Americas	24.0 (6.0–35.0)^**^	13	53.0 (40.0–61.0)	9
	Africa	5.8 (0.0–53.2)^*^	3	58.0 (53.9–62.0)	2
	Total	65.1 (32–80)^**^	52	40 (58.3–78)^*^	50

* *p < 0.05*,

** *p < 0.005*,

*** *p < 0.0005. P-values in the “Total” rows refer to the Kruskal Wallis tests assessing if there is a difference in AMR prevalence between all regions. The p-values in the other rows refer to the Wilcoxon rank sum tests comparing the prevalence in Europe with each other region*.

#### Azithromycin

The prevalence of resistance to azithromycin was highest in Europe in 2009 (6.7, IQR 2.3–17.3) and Africa in 2014 (11.0, IQR 10.0–83.1). The prevalence of azithromycin resistance was lower in Western Pacific (0.0, IQR 0.0–0.0, *p* < 0.05) compared to Europe in 2009.

#### Ciprofloxacin

Resistance to ciprofloxacin was highest in South East Asia at both time points (2009: 93.2%, IQR 83.7–96.7; 2014: 91.1%, IQR 76.4–99.1). Both these prevalences were higher than those from Europe (2009: 67.3%, IQR 43.3–79.2; 2014: 57.0%, IQR 34.7–67.5; both *p* < 0.05). In addition, the prevalence of resistance in 2009 was lower in the Americas (24.0%, IQR 6.0–35.0) and Africa (5.8%, IQR 0.0–53.2) than Europe (*p* < 0.05).

### Antimicrobial consumption

#### Cephalosporins

In 2000, 2008, and 2009, the consumption of cephalosporins was highest (and increased steadily) in Western Pacific (2000: median standard units/1,000 persons/year- 2000: 2071, IQR 1237–4686; 2008: 2657, IQR 1766–5601; 2013: 3237, IQR 2665–6991; Table 2, Figure 1). Compared to Europe, these higher consumption rates were only statistically significant in 2008 and 2013 (*p* < 0.05). Consumption was lowest in South East Asia in 2000 (418, IQR 154–418) but increased rapidly by 2008 (1450, IQR 750–2141) and 2013 (1568, IQR 1132–2707). Although consumption declined considerably over time, Japan's cephalosporin consumption was in the top three for 2000, 2008, and 2013 (Supplementary Table 1). In 2000, Japan's consumption (11 192) was nearly twice that of the second highest consumer (France: 5802) and 72 times that of the lowest consumer.

**Table 2:** Antimicrobial consumption by country reported in world regions.

		**2000**	***N***	**2008**	**N**	**2013**	***N***
Cephalosporins	Europe	1573 (779–2850)	22	1623 (590–2522)	22	1385 (656–2112)	22
	SE Asia	418 (154–418)*	3	1450 (750–2141)	4	1568 (1132–2707)	4
	W Pacific	2071 (1237–4686)	9	2657 (1766–5601)*	10	3237 (2665–6991)**	10
	Americas	1132 (526–1846)	10	1142 (814–2214)	10	1402 (921–2289)	10
	Africa	769 (769–769)	1	711 (711–711)	1	728 (728–728)	1
	Total	1644 (814–2522)*	45	1644 (814–2522)*	47	1570 (769–2754)*	47
Macrolides	Europe	2299 (1545–3053)	22	2206 (1449–2817)	22	1963 (1236–2390)	22
	SE Asia	559 (266–785)*	3	759 (579–1152)*	4	900 (711–1167)*	4
	W Pacific	2345 (1128–2855)	9	2042 (1512–2834)	10	2205 (1703–2884)	10
	Americas	728 (469–1366)**	10	775 (647–1330)**	10	998 (764–1521)**	10
	Africa	1720 (1720–1720)	1	1743 (1743–1743)	1	1870 (1870–1870)	1
	Total	1720 (895–2797)**	45	1612 (938–2645)*	47	1626 (1010–2257)*	47
Fluoroquinolones	Europe	936 (614–1246)	22	1290 (761–1757)	22	1211 (698–1620)	22
	SE Asia	715 (137–1238)	3	1330 (964–1732)	4	1116 (874–1553)	4
	W Pacific	590 (365–1110)	9	812 (466–1690)	10	1198 (457–1658)	10
	Americas	461 (355–943)	10	840 (684–1405)	10	1059 (809–1597)	10
	Africa	316 (316–316)	1	827 (827–827)	1	881 (881–881)	1
	Total	741 (383–1150)	45	968 (761–1690)	47	1183 (758–1600)	47

Consumption reported as number of standard doses per 1,000 population per year [median (interquartile range)] Data from IMS. *p < 0.05, **p < 0.005; P-values in the “Total” rows refer to the Kruskal Wallis tests assessing if there is a difference in antimicrobial consumption between all regions. The p-values in the other rows refer to the Wilcoxon rank sum tests comparing the consumption in Europe with each other region.

#### Macrolides

Macrolide consumption was highest in Europe in 2000 (2299, IQR 1545–3053) but this declined by 2008 and 2013. Although macrolide consumption increased in both the Americas and South East Asia between 2000 and 2013, it remained lower at all three times in these two regions than Europe (*p* < 0.05).

#### Fluoroquinolones

Fluoroquinolone consumption increased steadily in all regions between 2000 and 2008 but then declined slightly between 2008 and 2013 in Europe and SE Asia. Consumption was highest in Europe in 2000 but by 2013 was similar in Europe, SE Asia and Western Pacific.

### Correlation between antimicrobial resistance and consumption

At the level of WHO regions, the prevalence of AMR for the three antimicrobials was positively associated with the consumption rates of those antimicrobials in five out of six assessments (*r* = 0.37–0.94; Table 3).

**Table 3:** Pearson's correlation between consumption of antimicrobial and antimicrobial resistance by WHO world region.

	**2009**	**2014**
Ceftriaxone	0.88	0.94
Azithromycin	0.38	0.22
Ciprofloxacin	0.66	0.37

The association between antimicrobial resistance and consumption at the level of individual countries was positive in six out of six assessments. In four instances the positive associations were statistically significant (cephalosporins 2008: coefficient 0.0005 [95% CI 0.0002–0.0007] and 2013: coefficient 0.0003 [95% CI 0.0002–0.0004]; macrolides 2013: coefficient 0.0005 [95% CI 0.00002–0.001]; fluoroquinolones 2013: coefficient 0.02 [95% CI 0.006–0.031]; Table 4, Figure 1).

**Table 4:** Linear regression coefficients for association between antimicrobial consumption (2008 and 2013) and percent antimicrobial resistance for quinolones, macrolides and ceftriaxone (2009 and 2014; coefficient [95% confidence interval]).

**Antimicrobial**	**2009**	***N***	**2014**	***N***
Cephalosporins	0.0005 (0.0002–0.0007)^*^	40	0.0003 (0.0002–0.0004)^**^	34
Macrolides	0.00008 (−0.0007–0.0009)	27	0.0005 (0.00002–0.001)^*^	35
Fluoroquinolones	0.0006 (−0.0006–0.002)	40	0.02 (0.006–0.031)^*^	38

* *p < 0.05*,

** *p < 0.005; Regression analyses were performed with square root transformed AMR variables*.

### Correlation between incidence of NG and antimicrobial resistance/consumption

At the level of WHO regions, the incidence of Ng was positively associated with the consumption rates of those antimicrobials in 3 out of 6 assessments and (*r* = 0.48–0.85; Table 5), and negatively associated in 1 assessment (*r* = −0.63). The incidence of Ng was positively associated with AMR rates in 3 out of 6 assessments and (*r* = 0.38–0.90; Table 5), and negatively associated in 1 assessment (*r* = −0.45).

**Table 5:** Pearson's correlation between incidence of gonorrhea in 2012 and (a) antimicrobial resistance in 2014 (b) antimicrobial consumption in 2013 by WHO world region.

	**Women**	**Men**
a)
Ceftriaxone	0.90	0.38
Azithromycin	−0.45	0.60
Ciprofloxacin	0.26	−0.19
b)
Ceftriaxone	0.06	0.85
Azithromycin	0.48	0.34
Ciprofloxacin	−0.63	0.28

## Discussion

In this ecological analysis, we found that the consumption of cephalosporins was highest in Western Pacific in general and Japan in specific. Furthermore, at the level of regions and countries there was evidence of a positive association between volume of cephalosporin consumption and AMR.

The associations between macrolides/fluoroquinolone consumption and resistance whilst also positive were only statistically significant in 2014. At all three time points fluoroquinolone AMR was highest in South East Asia—the region with the highest or second highest fluoroquinolone consumption in 2000 and 2008. Macrolide AMR was highest/second highest in Europe in 2008/2013, whilst macrolide consumption in Europe was highest or second highest in 2000, 2008, and 2013.

There are a number of possible explanations for these findings. The prevalence of antimicrobials consumed by the general population may be correlated with the antibiotics used to treat Ng-associated STI syndromes and this may be the actual driver of AMR in Ng. This explanation is supported by a large body of evidence that has demonstrated that resistance to a particular antimicrobial in Ng typically follows soon after the introduction of that antimicrobial as recommended therapy for Ng (2, 19, 20). This explanation does not however explain why cephalosporin AMR emerged so explosively in Japan in the 2000s when Japan was using the same therapy for Ng as many other countries (cefixime) where AMR did not emerge as rapidly. An alternative explanation is that the volume of antimicrobials consumed in the general population plays a role in the genesis of certain forms of AMR in Ng as has been established in other bacteria (14–17). A further example of this phenomenon is the reported emergence of a gonorrhea strain that was resistant to cephalosporins in France in 2011, which at the time was the second largest consumer of cephalosporins (21).

This pharmacoecological theory of AMR posits that extensive antimicrobial exposure (including that used for STI treatment) in a population places a selection pressure on circulating Ng to acquire AMR via five mechanisms (9, 22, 23). One of these mechanisms is the acquisition of resistance-conferring-DNA from commensal bacteria (24). Treatment with antibiotics such as macrolides and cephalosporins results in an increase in the prevalence of resistance mutations in commensals from the pharynx, colo-rectum and elsewhere (25, 26). In the case of macrolides these changes can persist for up to 4 years (26). Ng is able to take up these resistance determinants (via transformation) (4, 27). It has been established via *in vitro* experiments and phylogenetic analyses that transformation with DNA from commensal pharyngeal *Neisseria* spp. was the likely method whereby Ng acquired resistance to cephalosporins in Japan (4, 20, 28, 29). Because ~90% of rectal and pharyngeal infections and the majority of cervical Ng infections are asymptomatic and persist for months, Ng is asymptomatic for the vast majority of its time circulating in populations (30, 31). These asymptomatic Ng infections would be under selection pressure to develop AMR to the antimicrobials most commonly used in the local populations and not just those used to treat symptomatic STIs (22, 32). Our findings are broadly compatible with this theory. In particular, the theory offers a parsimonious explanation for the correlations between antimicrobial use and resistance at regional and country levels. These include the high rates of cephalosporin resistance in Western Pacific, macrolide resistance in Europe and quinolone resistance in South East Asia. According to this theory, the problem of cephalosporin resistance of Ng could be productively viewed as being, to some extent, part of an allodemic of resistance to cephalosporins in a wide range of bacteria in the Western Pacific (33–35). The populations in the rural Australia and some parts of the Pacific Island Countries with extremely low rates of AMR in Ng and low antimicrobial consumption are also compatible with this theory (36–38). There are however also a number of countries and regions with a discordance between AMR and antimicrobial consumption. The high/low prevalence of ceftriaxone AMR/consumption in China in 2009 is one example. This outlier is however likely explained by an inaccurately high AMR prevalence estimate. A recent national surveillance of AMR report from China estimated that 10.8% of Ng isolates from 2013 to 2016 had ceftriaxone MICs ≥0.125 μg/ml (39). This is considerably lower that the estimate we used for 2009 (55.5% of isolates with an MIC >0.125 μg/ml).

Previous studies evaluating the ecological-level association between antimicrobial consumption and AMR have had divergent findings. In an ecological analysis of county level data from the United States 2005–2013, Kirkcaldy et al. did not find an association between antimicrobial consumption and emergence of AMR in Ng (12). A more recent analysis from the United states has, however, found that gonococcal azithromycin MICs were positively associated with seasonal variations in population macrolide consumption (40).

A complex interplay between a wide array of factors are likely responsible for the differential emergence and spread of AMR in Ng (27). These include: differences in Ng strain propensity to acquire AMR/compensatory mutations and spread (6, 41), variations in sexual network structure (10, 42), pattern of consumption of antimicrobials in core-groups (10), variations in the prevalence of sexual practices (such as anal sex which may facilitate access to resistance mechanisms in the rectal microbiome) (22, 42), interactions between pharmacokinetics of antimicrobials used and site of Ng infection (12, 27), local, regional, and intercontinental travel (43), local and national STI screening practices (12, 44, 45), differences in rates of non-prescription antimicrobial consumption (46), and variations in population microbiomes/resistomes (42). A recent systematic review summarized the risk factors associated with AMR in Ng in 24 studies (36). This review found that AMR is more common in MSM than heterosexual men as well as being higher in certain ethnic groups than others. We were unable to control for any of these other risk factors in our study. Further study limitations include the fact that the data is only until 2014, the small sample sizes and incomplete data for a number of countries. The analyses are all ecological and thus susceptible to the ecological inference fallacy. This is particularly problematic in our regional correlation analyses since we used all data points that were available for each time in each of the two databases. As a result, we were not always comparing data from the same countries within each region.

There may be inaccuracies in the AMR and antimicrobial consumption data. The IMS methodology misses non-reporting manufacturer direct sales. This may introduce a bias if this varies between countries (46, 47). Although this issue has not been assessed in all participating IMS countries, where it has been assessed, these omissions were found to constitute only 0.5% of all sales (47, 48). Overall the consumption estimates from IMS have been found to correlate closely with those from other estimates such as those from the European Surveillance of Antimicrobial Consumption Network (49). Furthermore, as far as we can establish, the IMS database provides the only source of harmonized data on global antimicrobial consumption (49). We used a 1-year time lag between antimicrobial consumption and AMR as this has been previously shown to provide the best fit to the emergence of penicillin and macrolide resistance in *Streptococcus pneumonia* (50). This time lag has not, however, been validated for Ng. Because changes in the resistome appear to persist for a longer time following macrolides than cephalosporins and quinolones, future studies could investigate if a shorter lag period should be used for cephalosporins (26, 50).

There has been a large increase in antimicrobial consumption in low and middle-income countries over the last 15 years (49). Antimicrobial consumption in India for example increased 65% between 2000 and 2015 (49). A large proportion of this increase was in cephalosporins (49). If population-level consumption of particular antimicrobials above a particular threshold increases the probability of AMR in Ng then more thought needs to put into monitoring this threat. More generally our study highlights the utility of programmes such as GASP/IMS that monitor AMR/antimicrobial consumption globally. Expanding the geographical and temporal coverage of these valuable resources would enable more detailed analyses that are able to control for confounders and thereby provide a clearer elucidation of the factors underpinning the emergence of AMR in Ng.

Finally our study further highlights the critical need to strengthen antibiotic stewardship programmes to limit exposure of Ng to antimicrobials and thereby delay the further emergence of resistance in Ng.

## Summary points

- Previous studies have found positive ecological associations between antimicrobial consumption and the prevalence of antimicrobial resistance
- We used susceptibility data from the WHOs Global Gonococcal Antimicrobial Surveillance Programme and antimicrobial consumption data from the IMS Health MIDAS database to assess if the same was true for *Neisseria gonorrhoeae*
- The assessments were carried out for ceftriaxone, azithromycin and ciprofloxacin at two time periods-−2008/9 and 2013/24
- In six out of six assessements the association between antimicrobial consumption and resistance was positive
- In the case of three assessments these associations were statistically significant.

## Author contributions

CK conceptualized the study. CK was responsible for the acquisition, analysis, and interpretation of data. TW provided GASP data. CK, JB, and TW played a role in writing, editing, and approving the final version.

### Conflict of interest statement

The authors declare that the research was conducted in the absence of any commercial or financial relationships that could be construed as a potential conflict of interest.

## Supplementary material

Supplementary Table 1 Antimicrobial resistance and consumption by country.
